# Vitamin D effects on *Chlamydia trachomatis* infection: a case-control and experimental study

**DOI:** 10.3389/fcimb.2024.1366136

**Published:** 2024-04-18

**Authors:** Sijia Liu, Tianwei Zhao, Quanzhong Liu

**Affiliations:** ^1^ Institute of Sexually Transmitted Diseases, Tianjin Medical University General Hospital, Tianjin, China; ^2^ Department of Dermatology, Beijing Ditan Hospital, Capital Medical University, Beijing, China

**Keywords:** vitamin D deficiency, *Chlamydia trachomatis*, antibiotics, vitamin D supplementation, calcitriol

## Abstract

**Introduction:**

Vitamin D deficiency is the most common nutritional deficiency worldwide. Chronic vitamin D deficiency causes immune system dysfunction, which increases susceptibility to pathogens such as bacteria, especially intracellular parasites, and viruses. *Chlamydia trachomatis* (C. t) is an obligate intracellular parasitic bacterium that causes a variety of sequelae. We speculated that vitamin D might be associated with C. t infection. This study aimed to address this gap in knowledge by investigating the relationship between vitamin D and C. t infection using both *in vitro* and *in vivo* models.

**Methods and results:**

The addition of calcitriol to McCoy cell culture *in vitro* delayed and reduced the quantity and volume of inclusions compared to the control group. Macrophages of peritoneally lavaged mice co-cultured with McCoy decreased the infection rate and delayed the appearance of inclusions. In mice models of vitamin D deficiency, mice in the VD-group exhibited more severe genital tract inflammation and a longer duration of infection after inoculation with C. t in the genital tract. Supplementing these mice with vitamin D3 during treatment enhanced the therapeutic effect of antibiotics. We also conducted a case-control study involving 174 C. t-positive patients (95 males and 79 females) and 380 healthy volunteers (211 males and 169 females) aged 20–49 from January 2016 to March 15, 2017. Serum 25-(OH)D concentration was measured by assessing morning fasting blood samples of healthy volunteers and C. t-positive patients 1 day before antibiotic treatment and the next day after one course of treatment. The patients were followed up for 1 month and evaluated for recovery. The results showed that vitamin D deficiency was a risk factor for C. t infection and treatment failure.

**Conclusion:**

In summary, findings from experimental and clinical studies indicate a close association between vitamin D levels and C. t infection and treatment outcomes. Given the affordability and safety of vitamin D, both healthy individuals and patients should focus on vitamin D intake. Vitamin D supplementation could enhance treatment success and should be used as an adjunctive therapy alongside antibiotic therapy for C. t infections, pending confirmation in larger, prospective, randomized controlled trials.

## 1 Introduction

*Chlamydia trachomatis* (Chlamydia. t, C. t) infection is a common sexually transmitted disease globally, with an incidence of approximately 131 million cases per year (Newman et al., 2015). Over 50% of patients are asymptomatic, facilitating the widespread transmission of the disease and increasing the risk of late complications. C. t irreversible damages the urogenital system, resulting in various outcomes, including (but not limited to) ectopic pregnancy, pelvic inflammatory disease, preterm labor, hydrosalpinx, tubal-induced infertility, chronic pelvic pain in females, alongside epididymitis, testicular pain, and infertility in males (Brunham and Rey-Ladino, 2005; Oakeshott et al., 2010).

C. t, an obligate intracellular parasitic bacterium, contains various pathogen-associated molecular patterns on its surface, including LPS, heat shock proteins, lipoproteins, and pORFs. Immune cells possess numerous pattern recognition receptors, notably toll-like receptors (TLRs) and NOD-like receptors, which recognize C. t infections, activating innate immunity and subsequently stimulating specific T cells. LPS is a potent ligand for TLR4 and TLR2, which recognizes live elementary bodies (EBs) and pathogen-associated molecular patterns and can induce pro-inflammatory and anti-inflammatory pathways (O'Connell et al., 2006). NLRs are cytoplasmic receptors that play important roles in microbial recognition and innate defense.

Vitamin D deficiency is the most common nutritional deficiency worldwide, affecting approximately 30% of children and 60% of adults (Daly et al., 2012). Vitamin D deficiency is also common in Chinese individuals, with 83% of Chinese adults having 25-(OH)D concentrations less than 30 ng/mL, of which 32.7% were vitamin D insufficient (20–29 ng/mL), 41.9% were vitamin D deficient (10–19 ng/mL), and 8.4% were severely vitamin D deficient (10 ng/mL) (Jiang et al., 2020). A study involving 1985 healthy pregnant Chinese women showed that 74.9% had vitamin D deficiency (Yun et al., 2017). In China, 35.7% of the elderly population aged ≥80 years had insufficient vitamin D levels, with 41.3% being vitamin D deficient (Liu et al., 2020). The main cause of vitamin D deficiency is insufficient exposure to the sun. Vitamin D is converted by 25-hydroxylase to 25-hydroxyvitamin D (25-(OH)D), which is catalyzed by 1-alpha-hydroxylase to 1,25-dihydroxvitamin D3 (1,25-(OH)2D3, calcitriol). Calcitriol is the most active vitamin D metabolite and a potent immunomodulator necessary for combating pathogens (Zdrenghea et al., 2017). Most immune cells, including B cells, T cells, neutrophils, macrophages, and dendritic cells, express 1-α-hydroxylase and VDRs (Baeke et al., 2020), and increased intracellular synthesis of calcitriol and VDRs, stimulated by TLR-4 signaling, can further activate immune cells and increase the production of antimicrobial peptides and neutralizing antibodies (Aygun, 2020; McGregor et al., 2020; Chauss et al., 2022). Chronic vitamin D deficiency leads to immune system dysfunction, which increases susceptibility to pathogens such as bacteria, especially intracellular parasites, and viruses (Ismailova and White, 2022). Consequently, we hypothesized a potential association between vitamin D and C. t infection.

Therefore, this study aimed to determine the relationship between vitamin D and C. t infection *in vitro*, assess the effect of vitamin D deficiency on genital tract C. t infection in animal models, and analyze the association between human serum 25-(OH)D levels, C. t infection in the urogenital tract, and antibiotic treatment effects through case-control studies.

## 2 Materials and methods

### 2.1 Acquisition and purification of murine peritoneal macrophages

BALB/C mice aged 6–8 weeks (purchased from the Institute of Laboratory Animal Sciences) were anesthetized and euthanized. The abdominal skin was cut to expose the abdominal wall, which was sterilized with 75% alcohol. A pre-cooled complete culture medium (10 mL) was injected into the abdominal cavity, followed by gentle massage of the abdomen for 2 min and repeated aspiration to obtain a cell suspension. The abdominal wall was then stretched to form a pouch from which the cell suspension was retrieved. The cell suspension was centrifuged at 4 °C and 1000 rpm for 20 minutes, and the supernatant was discarded. The cells were resuspended in a complete culture medium, adjusted to a concentration of 10^6^/mL, and added to a 24-well plate. Macrophages were isolated using the differential adhesion method. The plate was then placed in the incubator for 2–3 h, discarding the supernatant, washing twice with Hank’s solution, and adding the culture medium for continued growth for 24 h to obtain mouse macrophages. McCoy cells were added for subsequent experiments.

### 2.2 Culture of McCoy cells and C. t

McCoy cells (purchased from the Institute of Dermatology, Chinese Academy of Medical Sciences, and stored in the Tianjin Institute of Sexually Transmitted Diseases) were cultured to a dense, single-layer state for cell passage. The cells were digested using trypsin EDTA solution, and the resulting cell suspension was prepared by repeatedly blowing with a complete culture medium. The cell suspension was added to 6-well plates and shaken to evenly distribute the cells. After culturing in an incubator at 37 °C and 5% CO_2_, the cells formed dense monolayers after 18–24 h.

The cells were treated with phosphate buffer saline solution supplemented with 30 μg/mL DEAE-D (diethylaminoethyl-dextran) for 20 min. Subsequently, 1 mL of DMEM medium and C. t E/UW5/Cx standard strain (provided by the Chlamydia Research Center of the University of Maryland, USA, and preserved in the Tianjin Institute of Sexually Transmitted Diseases) was added, placed in the incubator for 30 min, centrifuged for 1 h at 32 °C and 1500 rpm, and incubated for another 30 min before replacing the infection solution (complete culture solution containing 1 μg/mL cycloheximide). Offspring EBs were collected and stored after 48 h of cultivation (Scidmore, 2005).

### 2.3 Plotting the growth curves of C. t E

After replacing the infection solution, the inclusions were stained and counted every 2–4 h to plot growth curves. Inclusions were observed using iodine staining. After cell fixation, a 4–5-fold dilution of Lugol’s solution was added to cover the cell and stained for 1 min, with the inclusions stained brown.

### 2.4 Establishment of a mouse vitamin-deficiency model

Vitamin D-deficient mouse models were established following the method of James et al (Fleet et al., 2008), and serum 25-(OH)D levels were reduced to 25–40 nmol/L. All mice were allowed free access to water and food. Twenty-five mice in the VD- group were fed a vitamin D-deficient diet (containing less than 25 IU VD3/kg) under incandescent lighting, while 15 mice in the VD+ group were fed a high vitamin D diet of 50000 IU VD3/kg and exposed to sunlight. The light-dark cycle was 12 hours. Following this diet and light exposure for 8 weeks, angular venous blood was collected from the mice. ELISA (Roche, Switzerland) was used to determine the serum 25-(OH)D levels.

### 2.5 Establishment of a mouse reproductive tract infection model of C. t

According to the previous research method (Wang et al., 2006), mice with satisfactory serum vitamin D levels were selected, and 2.5 mg of progesterone injection was injected intramuscularly 7 days prior to the infection. A sterile male urethral swab was inserted into the cervical opening for dilation, and after inoculating 50 μL of C.t E in the vagina, gelatin sponges stained with C.t E fluid were inserted into the vagina. The mice were inverted for 2 h to prevent fluid flowing. Secretions were taken by swabbing and used for subsequent culture, immunofluorescence, and IFU (inclusion forming units) assays.

### 2.6 Immunofluorescent assay detection of C. t

The samples were covered with staining solution (Chlamydia trachomatis direct immunofluorescence detection kit: GenStar Biological), incubated for 15 min at room temperature, rinsed with ddH2O for 10 s, and observed under a fluorescence microscope.

### 2.7 PCR method for detecting C. t

The mouse secretion sample was centrifuged in 1.5-mL tubes at 12000 rpm for 20 min. After discarding the supernatant, lysis solution (50 mmol/L KCl, 2.5 mmol/L MgCl2, 10 mmol/L Tis-HCl (pH 8.3), 0.1 g/L gelatin, 0.45% NP-40, 0.45% Tween 20, and 200 mg/L protease K) was added and incubated at 55 °C for 1 h and boiled for 15 min to inactivate protease K, followed by centrifugation at 12000 rpm for 3 min and collection of the supernatant.

PCR primers were designed as follows: forward primer 5’GGACAAATCGTATCTCGG3’; reverse primer 5’GAAACCAACTCTACGCTG3’. PCR amplification was carried out, and the amplification products were detected through 2% agarose gel electrophoresis. Positive specimens will exhibit clear and specific bands at 517 bp.

### 2.8 Inclusion and exclusion criteria for clinical studies

From November 1, 2016, to March 15, 2017, we enrolled 174 patients (95 males and 79 females) with C. t positive and 380 healthy volunteers (211 males and 169 females) at our hospital. According to the case-control study design, frequency matching was performed based on sex and age, and when the number of matched samples was large, those with the most similar body mass index (BMI) and education level were preferred, and unmatched samples were discarded. This ensured that controls for infected and non-cured cases were selected from healthy and cured populations, respectively. Finally, infected patients and healthy controls (161 cases each) and non-cured and cured patients (41 cases each) were chosen.

Inclusion criteria: (1) Age 20–49 years; (2) Settled in Tianjin for more than 1 year; (3) 18.5 ≤ BMI <28.0 kg/m^2^; (4) Good compliance. Exclusion criteria: (1) Individuals with respiratory, cardiovascular, digestive, endocrine, hematological and autoimmune diseases, and malignant tumors; (2) Individuals who have undergone surgery, blood transfusion or donation, taken glucocorticoids or immunosuppressive drugs within 6 months, used antibiotics or vitamin D supplements within 1 month, and experienced fever or colds within 2 weeks; (3) Pregnant and lactating women; (4) Healthy individuals excluding those with symptoms of urethritis or cervicitis; (5) C. t positive patients excluding those with other sexually transmitted diseases; (6) When comparing the difference between the cured group and the non-cured group, those who refused treatment, use medication incorrectly, have sexual contact during treatment, or withdraw from the study should be excluded. Participants were screened via questionnaires, physical examinations, and laboratory tests, and their basic information was recorded. All enrolled individuals were informed about the necessary tests and serum usage and provided informed consent. This study was approved by the Ethics Committee of Tianjin Medical University General Hospital.

### 2.9 Patient sample collection and testing

All C. t-positive patients underwent genital tract sampling before and 1 month after completing 1 week of antibiotic treatment. Sampling involved collecting urethral secretions from males and cervical secretions from females after 1 h of urination. Samples were tested using the diagnostic kit (Roche, Switzerland) for C. t. Simultaneously, patients were screened for other sexually transmitted diseases using the HIV antigen and antibody diagnostic kit (Livzon, Zhuhai, China), the passive particle agglutination test for detecting *Treponema pallidum* antibody (SERODIA-TPPA, Japan), and the Mycoplasma identification susceptibility test kit (encode, Zhuhai, China).

Fasting venous blood (5 mL) was collected from patients 1 day before and the morning after one course of treatment and from healthy volunteers. Serum was obtained after centrifugation, and 25-(OH)D levels were measured using an ELISA kit (Roche, Switzerland).

### 2.10 Statistical analysis

Statistical analysis was conducted using SPSS 25.0 software. In the experimental research, the differences in the number of inclusions among different groups were analyzed using independent sample T-tests, and *P* < 0.05 indicated significant differences.

In the case-control study, chi-square tests were used to balance the general demographic characteristics of the infected patients and healthy controls. Non-conditional logistic regression was used to calculate the odds ratio (OR) value and its 95% confidence interval (95% CI) to assess the association between 25-(OH)D levels and decreased risk of C. t infection and disease progression. Independent sample T-tests and non-parametric tests were used for measurement data analysis, while the chi-square test was used for count data. *P* < 0.05 indicated statistically significant differences.

## 3 Results

### 3.1 Effect of calcitriol on the infection rate of C. t *in vitro*

**Figure 1:**
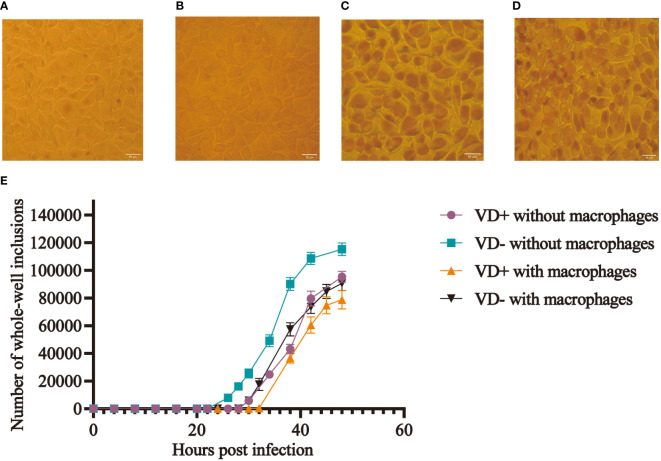
Effect of calcitriol on the infection rate of C. t *in vitro*. More inclusions were observed in the control wells **(A)**, and occasional inclusions were observed in the experimental wells **(B)** at 28 h post-infection. Inclusions **(C)** in the control wells and smaller sizes of inclusions **(D)** in the experimental wells at 48 h post-infection. **(E)** Growth curves of C. t E on McCoy cells with and without macrophages in the control and experimental groups.

The McCoy cells from the calcitriol experimental group were pre-cultured with calcitriol at a concentration of 10^-7^ mol/L for 24 h. The same concentration of calcitriol was added to both DMEM and infection culture medium during the inoculation of the C. t E standard strain. The number of whole-well inclusions was counted at different time points after fixation and iodine staining, and growth curves were plotted (**Figure 1E**). In the control group, inclusions first appeared 20 h after infection, while in the experimental group, they appeared after 26 h, with an average delay of 6 h (**Figures 1A, B**). The infection rate decreased by about 15% (*P* < 0.05) after 48 h. Moreover, the volume of inclusions in the experimental group was smaller than that in the control group (**Figures 1C, D**).

Mouse macrophages were co-cultured with McCoy cells and inoculated with C. t, and mice in the experimental group were treated with calcitriol. Results indicated that inclusions appeared 32 hours post-infection in the experimental group, compared to 26 hours in the control group, representing a delay of approximately 6 hours in the experimental group. At the 48th hour, the final infection rate decreased by approximately 9.11% (*P* < 0.05) (**Figure 1E**).

### 3.2 Effect of 25-(OH)D level on C. t infection in the urogenital tract of mice

Forty BALB/C female mice were randomly divided into two groups: 25 mice in the VD- group and 15 mice in the VD+ group. After 8 weeks of feeding, the average serum 25-(OH)D levels of mice in the VD- and VD+ groups were 33.66 ± 5.36 nmol/L and 650.47 ± 83.53 nmol/L, respectively (**Supplementary**-**Table S1**). The vitamin D-deficient mouse model was successfully modeled.

The genital tract of mice was infected with C. t E. Vaginal secretion specimens were collected at weeks 1–12 post-infection. C. t in mouse vaginal secretions could be detected using IFU, PCR, and direct immunofluorescence assay. To determine the optimum assays, these three methods were employed to assess the positivity of the mice’s secretions one week after infection.Vaginal secretion samples were incubated in McCoy cells for 48–72 h, followed by iodine staining to detect inclusions. For negative specimens, continuous culturing was performed until the third generation. The positive rates after 7 days of infection were 68% (17/25 mice) in the VD- group and 66.7% (10/15 mice) in the VD+ group.

To avoid false negative results, PCR was performed on mice samples, which showed that all samples were positive.

Direct immunofluorescence was used to detect C. t on secretion smears. Under the fluorescence microscope, epithelial cells appeared red, while typical EBs appeared as bright dot-like substances. In the high-power field of view, EBs emitted uniform, medium-intensity fluorescence with smooth and circular edges. Usually, the result is considered positive when there are more than 10 EBs in a smear. The immunofluorescence results of all mice in the first week after infection were positive.

Among these three methods, the cell culture assay was prone to false-negative results and had lower sensitivity. The PCR method may detect dead residual bacterial cells, resulting in false-positive results. The direct immunofluorescence method had appropriate specificity and sensitivity. Hence, in subsequent experiments, the direct immunofluorescence method was chosen to detect C. t in the reproductive tract of mice.

**Figure 2:**
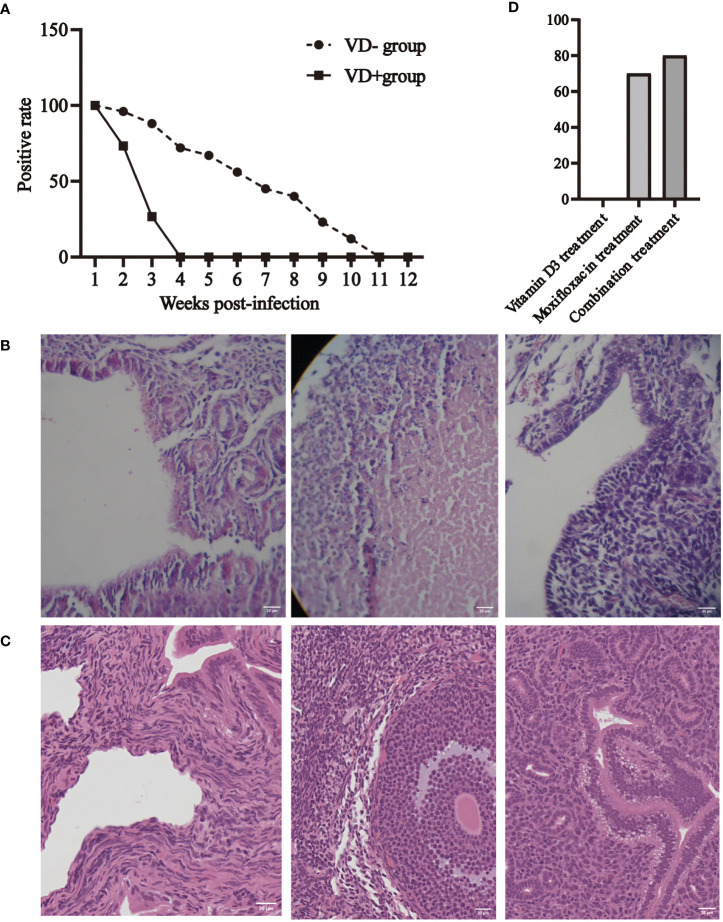
Effect of 25-(OH)D levels on C. t infection in the urogenital tract of mice. **(A)** Positive rates of immunofluorescence assay at different times after infection in VD+ and VD- groups. **(B)** Chronic inflammation with a high degree of lymphocyte infiltration characterizes the pathological alterations in the fallopian tubes, ovaries, and uterine horns of mice in the VD- group. **(C)** Pathological biopsy of VD+group mice. **(D)** The conversion rates after treatment with vitamin D3, moxifloxacin, and combination therapy.

We performed a direct immunofluorescence assay on smears from weeks 1–12 and simultaneously observed the characteristics of vaginal secretions (**Figure 2A**). The results showed that all mice were positive in the first week after infection, indicating successful modeling. Mice in the VD- group remained positive after the tenth week of infection, whereas mice in the VD+ group became negative from the fourth week of infection. The direct immunofluorescence staining of secretion smears could be seen in the **Supplementary Figure S1**.

In the first week post-infection, both VD+ and VD- mice showed a large amount of clear vaginal secretions. Mice in the VD- group showed a large amount of yellow, sticky secretions from the second week, which gradually turned into copious clear secretions and lasted until the tenth-week post-infection. Mice in the VD+ group had excessive and clearer vaginal secretions in the second week, followed by a gradual decrease. After the fourth week, the vagina became dry, with no significant secretions.

After 12 weeks of infection, the uterus, fallopian tubes, and ovarian tissues were removed from some mice in the VD- and VD+ groups, and hematoxylin & eosin staining was performed to evaluate inflammation. The uterus, fallopian tubes, and ovarian tissues of mice in the VD- group showed extensive lymphocyte infiltration, indicating chronic inflammation (**Figure 2B**). No significant change was observed in the VD+ group (**Figure 2C**).

### 3.3 Effect of 25-(OH)D on antibiotic efficacy *in vivo*

Thirty BALB/C female mice were used to create a vitamin D deficiency model. C. t E infection was performed after 8 weeks of feeding. One week after infection, secretions were collected for a direct immunofluorescence assay, confirming successful modeling as all samples tested positive. These mice were randomly divided into three groups and administered 30 mg/kg moxifloxacin, 250 IU/(kg.d) vitamin D3, and moxifloxacin combined with vitamin D3 by gavage for 7 days. Immunofluorescence testing was conducted again at the end of the treatment to determine positivity. The results showed that all mice in the vitamin D3 therapy group tested positive, while seven mice in the moxifloxacin treatment group and eight mice in the combination treatment group tested negative. The conversion rates were 0%, 70%, and 80% in the vitamin D, moxifloxacin, and combination treatment groups, respectively (**Figure 2D**).

### 3.4 The basic characteristics of participants

**Figure 3:**
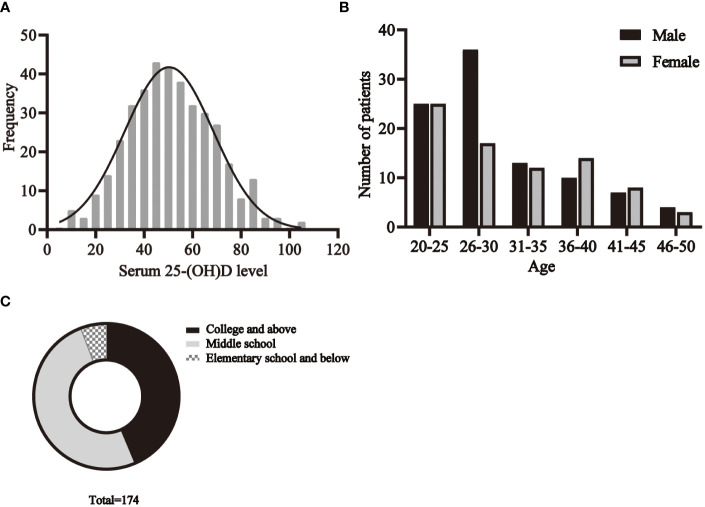
The basic characteristics of participants. **(A)** The distribution of serum 25- (OH)D levels in healthy individuals. The distribution of age **(B)**, education **(C)** among patients.

The basic characteristics of all participants are shown in **Table 1**. A total of 174 C. t positive patients and 380 healthy volunteers aged 20-49 years were recruited. The distribution of serum 25- (OH)D levels in healthy individuals is shown in **Figure 3A**.

**Table 1:** Serum 25-(OH)D level of the total population(nmol/L).

Age	Group	Gender			Cases	`x ± s
20-39	Healthy control	Male			180	53.72 ± 18.00
		Female			126	45.42 ± 16.08
	Infected patient	Male	Before treatment	Total	81	40.10 ± 17.93
			After treatment	Cured patient	54	41.32 ± 17.24
				Non-cured patient	18	30.50 ± 14.53
		Female	Before treatment	Total	67	35.71 ± 19.99
			After treatment	Cured patient	37	41.37 ± 21.03
				Non-cured patient	22	29.47 ± 16.66
40-49	Healthy control	Male			31	54.77 ± 15.66
		Female			43	56.06 ± 19.74
	Infected patient	Male	Before treatment	Total	14	42.08 ± 25.66
			After treatment	Cured patient	8	44.04 ± 27.96
				Non-cured patient	4	25.29 ± 9.04
		Female	Before treatment	Total	12	45.98 ± 24.34
			After treatment	Cured patient	5	54.03 ± 24.09
				Non-cured patient	6	30.24 ± 18.43

The age distribution of patients is shown in the **Figure 3B**. The results showed that among the C.t infected individuals, the highest percentage was in the age group of 20-30 years, followed by the age group of 31-40 years, while the 45-50 years old accounted for the least. Taking gender into account, it could be found that, in most situations, the proportion of men and women is nearly equal, but the number of infected men between 26 and 30 years old is much higher than that of women in this age group.

The education status of the patients is shown in **Figure 3C**. There were 76 people with college or higher education, 88 with middle school education, and 10 with elementary school education or less.

### 3.5 Relationship between C. t infection and 25-(OH)D levels

**Figure 4:**
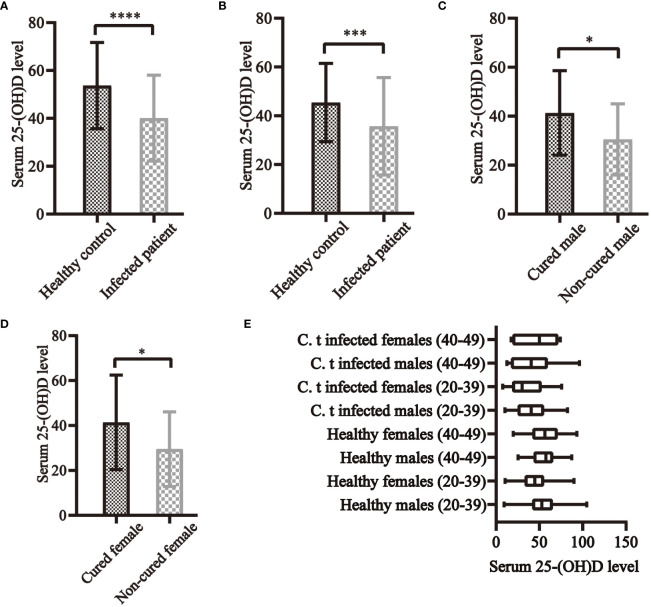
Serum 25-(OH)D levels and C. t infection in clinical research. In both males **(A)** and females **(B)** aged 20–39 years, serum 25-(OH)D levels were higher in healthy controls than in C. t-infected patients, all of which differed significantly. In both infected males **(C)** and females **(D)** aged 20–39 years, serum 25-(OH)D levels were all higher in cured than in non-cured patients, with statistically significant differences. **(E)** The levels of serum 25-(OH)D in each group of the population. * P<0.05, *** P<0.001, **** P<0.0001.

Considering sex, age, BMI, and education, 161 infected patients and 161 healthy controls were included to determine the relationship between C. t infection status and 25-(OH)D levels. Non-conditional logistic regression showed a moderate association between 25-(OH)D deficiency and C. t infection (OR = 2.281, 95% CI: 1.438, 3.619) (**Table 2**), suggesting that a decrease in serum 25-(OH)D levels may increase the risk of urogenital tract C. t infection. The 25-(OH)D levels in infected males aged 20–39 (81 individuals) years were 40.5 nmol/L lower than that in healthy males (180 individuals) 52.71 nmol/L, and the difference was statistically significant (*P* < 0.01) (**Figure 4A**). The 25-(OH)D level was significantly lower in C. t-positive females (n = 67, aged 20–39 years) at 30.11 nmol/L compared to that of 44.7 nmol/L in healthy females (n = 126) at 44.7 nmol/L (*P* < 0.01) (**Figure 4B**). In males aged 40–49 years, the mean 25-(OH)D concentration was higher in healthy males (31 individuals) than that in infected males (14 individuals), while the mean 25-(OH)D concentration was higher in healthy females (43 individuals) than that in infected females (12 individuals); however, these differences were not statistically significant (all *P* > 0.05).

**Table 2:** The relationship between serum 25-(OH)D level and C. t infection.

25-(OH)D level(nmol/L)	Infected patient n (%)	Healthy control n (%)	OR (95%CI) value	OR (95%CI)value ^a^
>50	47(29.2)	78(48.4)	1.000	1.000
≤50	114(70.8)	83(51.6)	2.279(1.440,3.608)	2.281(1.438,3.619)

a: Adjusted education and BMI.

### 3.6 Relationship between serum 25-(OH)D levels and duration of C. t infection

Forty-two cases, each for non-cured and cured patients, were included. 25-(OH)D deficiency was strongly associated with treatment failure (OR = 7.266, 95% CI: 2.551, 21.036) (**Table 3**), indicating that reduced serum vitamin D levels may lead to treatment failure and prolonged duration of infection. The 25-(OH) D level was significantly lower in non-cured males aged 20–39 years (n =18) at 30.50 ± 14.53 nmol/L than in cured males (n = 54) at 41.32 ± 17.24 nmol/L (*P* < 0.05) (**Figure 4C**). The 25-(OH) D level in non-cured females aged 20–39 years (n=22) was 29.47 ± 16.66 nmol/L, lower than that in cured females (n=37) at 41.37 ± 21.03 nmol/L, with a statistically significant difference (*P* < 0.05) (**Figure 4D**). In the 40–49 years age group, the mean 25-(OH)D concentration was higher in cured males (n=8) and females (n=5) than that in non-cured males (n=4) and females. However, these differences were not statistically significant (all *P* > 0.05).

**Table 3:** The relationship between serum 25-(OH)D level and duration of C. t infection.

25-(OH)D level(nmol/L)	Non-cured n (%)	Cured n (%)	OR (95%CI) value	OR (95%CI) value^a^
>50	8(19.0)	25(59.5)	1.000	1.000
≤50	34(81.0)	17(40.5)	6.250(2.331,16.758)	7.266(2.551,21.036)

a: Adjusted education and BMI.

### 3.7 Relationship between sex, serum 25-(OH)D levels, and cure rates

In individuals aged 20–39 years, 25-(OH)D levels were significantly higher in males than in females in the healthy population (*P* < 0.01). In the infected population, the mean concentration of 25-(OH)D in males was higher than in females, and the difference was not statistically significant. The cure rate in males (75.00%) was higher than in females (62.71%), with no statistically significant difference. No statistically significant difference was observed in these three indicators in individuals aged 40–49 years (**Figure 4E**).

### 3.8 Distribution of vitamin D deficiency in healthy individuals.

**Figure 5:**
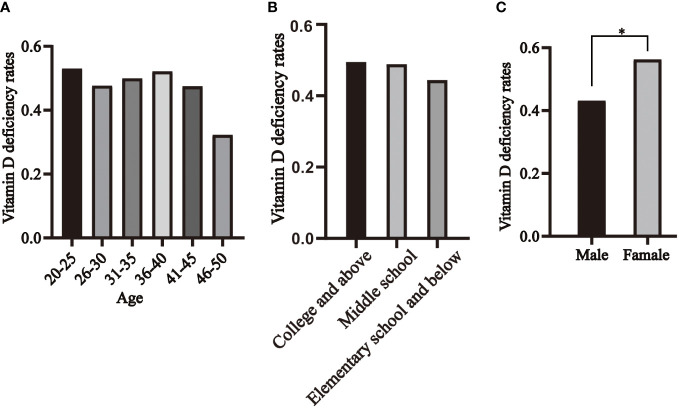
Distribution of vitamin D deficiency in healthy individuals, segmented by age **(A)**, educational levels **(B)** and genders **(C)**. * P<0.05.

We categorized the healthy population into six age groups, and the prevalence of vitamin D deficiency in healthy individuals was 53%, 48%, 50%, 52%, 48%, and 32% in the 20-25, 26-30, 31-35, 36-40, 41-45, and 46-50 age groups, respectively, with no statistically significant difference (**Figure 5A**). The prevalence of vitamin D deficiency was 49% in college and above, 49% in middle school, and 44% in elementary school and below, with no statistically significant difference (**Figure 5B**). 43% of men and 56% of women were vitamin D deficient, with the prevalence significantly higher in women than in men (**Figure 5C**).

## 4 Discussion

Numerous studies have reported a correlation between vitamin D and infectious diseases. Vitamin D supplementation could reduce the severity and risk of death in individuals infected with COVID-19 (Shah et al., 2022). In a meta-analysis involving 11 randomized controlled trials and 5660 patients, vitamin D exhibited protective effects against respiratory tract infections, and daily administration demonstrated superior protective effects compared to one-time administration (Bergman et al., 2013). Vitamin D also significantly affected Helicobacter pylori infections, with one study showing that vitamin D3 eradicated H. pylori *in vivo* and *in vitro*, including antibiotic-resistant strains (Hu et al., 2019). In this study, we explored the relationship between vitamin D and C. t infection and found that the addition of calcitriol to the cell culture medium delayed the appearance of inclusions and reduced their number and volume compared to the control group without calcitriol. In animal experiments, vitamin D-deficient mice had longer duration of infections and more severe inflammation of the reproductive tract. Administering vitamin D3 to infected mice during antibiotic treatment could improve the therapeutic efficacy. Analysis of 25-(OH)D levels in 174 C. t-positive patients and 380 healthy volunteers revealed that human urogenital tract C.t infections and disease progression were associated with vitamin D deficiency. Reduced serum 25-(OH)D levels may increase the risk of infection and decrease C. t negative conversion rates.

He et al. observed that adding vitamin D while cultivating C. t in Hela cells could reduce the infection rate (He et al., 2013). We used mouse epithelial McCoy cells for C. t E culture and obtained similar results. C. t initiates various processes such as internalization, maintenance of inclusion stability, and departure from the host cell by manipulating the host cytoskeleton (Caven and Carabeo, 2019). Upon contact with epithelial cells, C. t binds to the host cell’s surface and triggers endocytosis by secreting effector proteins into the cytoplasm via the type III secretion system (Birkelund et al., 1994; Clifton et al., 2004, Clifton et al., 2005). The addition of calcitriol to the cell culture medium alters the expression of cytoskeletal proteins such as tubulin, vimentin, tropomyosin chain, and vinculin (He et al., 2013), interfering with Chlamydia’s manipulation of the cytoskeleton and influencing inclusion formation, expansion, and stability. Autophagy, a key defense mechanism, involves autophagosomes recognizing intracellular pathogens for lysosomal degradation (Travassos et al., 2010). Autophagy in host cells is closely related to the life cycle of C. t and inhibits its development (Al-Younes et al., 2011; Al-Zeer et al., 2013). Calcitriol acting on eukaryotic cells can stimulate autophagy and eliminate pathogens. These mechanisms may be the reason for reduced inclusion formation, volume, and delayed appearance after calcitriol intervention.

Co-culturing McCoy cells with mouse macrophages and infecting them with C. t prolonged the formation of inclusions compared to the McCoy cell culture alone. Macrophages, abundant in pattern recognition receptors including TLRs and NOD-like receptors, can recognize pathogen-associated molecular patterns on the surface of EBs, activate downstream signaling pathways, and induce the expression of various cytokines, chemokines, and growth factors, such as IL-6, IL-8, TNF-α, IFN-α, and IFN-β (Belay et al., 2002; Shi and Pamer, 2011; Packiam et al., 2015). Thus, with or without calcitriol, the presence of macrophages during C. t culture could prolong inclusion appearance time and reduce the number of inclusions. After adding calcitriol, McCoy cell culture alone reduced the number of inclusions by 15%, whereas co-culture only resulted in a 9.11% reduction, probably because immune cell activation relies mainly on the stimulation of TLR-4 signaling, which increases the synthesis of intracellular calcitriol and VDRs within cells rather than calcitriol uptake in the interstitial fluid (Carlberg, 2023). Consequently, adding calcitriol to the cell culture medium had a limited effect on macrophage-mediated inhibition of C. t.

When the reproductive tract is invaded by C. t, innate immunity is the first line of defense. Epithelial cells first form a barrier that hinders the pathogen and its products from entering the submucosa. Pattern recognition receptors on epithelial cells can recognize EBs and activate leukocytes while recruiting monocytes and macrophages into the genital tract (O'Neill, 2002). These immune cells activate innate immunity, with antigen-specific IFN-γ-secreting CD4+ and CD8+ T cells infiltrating infected tissues to control and eliminate infection (Ziklo et al., 2016). The synthesis of calcitriol and VDRs within immune cells after pathogen recognition is crucial for activating immune cells and the production of antibodies and antimicrobial peptides. Vitamin D response elements (VDREs) are close to the transcription start sites of two genes encoding the antimicrobial peptides (AMPs), cathelicidin antimicrobial peptide (CAMP/LL37), and β-defensin 2 (DEFB2/DEFB4/HBD2) (Wang et al., 2004). AMPs can directly battle invading bacteria, fungi, and viruses while also disrupting the integrity of pathogen membranes. CAMP functions by participating in the immunological responses of immune cells. Epithelial cells, macrophages, neutrophils, and monocytes are capable of inducing CAMP expression in the presence of calcitriol (Takahashi et al., 2002; Wang et al., 2004). Johanna Gyll et al. found that salivary LL37 levels were positively correlated with serum vitamin D levels and that vitamin D deficiency increases caries risk (Gyll et al., 2018). The active form of CAMP, LL37, also has DNA-binding properties that augment signaling through TLR3, TLR7, or TLR9 to enhance interferon responses (Scheenstra et al., 2020). In an antiviral immune response mediated by calcitriol, LL37 binds to viral dsRNA and contributes to its recognition by PRR TLR3 (Singh et al., 2014). He et al. found that VDRs play an important role in C. t infection, with VDR gene knockout mice showing more severe genital infection and a slower clearance rate (He et al., 2013). To simulate human vitamin D deficiency, we employed a vitamin D-deficient mouse model and discovered that vitamin D-deficient mice exhibited slower resolution of C. t E vaginal tract infections and more severe inflammation. After 7 days of gavage of moxifloxacin and vitamin D3 in vitamin D-deficient mice, we observed that vitamin D alone was insufficient for eliminating C. t infections, but combined antibiotic administration improved antibiotic efficacy.

We recruited 174 C. t-positive patients and 380 healthy volunteers in the clinic. We first analyzed the distribution of age, gender, and educational background of patients, and the results showed that patients were mainly between 20 and 30 years old, followed by 31 and 45 years old, and the proportion of patients over 45 years old was very low. The highest prevalence was usually reported among young sexually active people, and the peak age of infection was 20-29. This trend is also reflected in the research of Changchang Li et al (Ng et al., 2014; Li et al., 2021). and Brian E. et al. Among all patients, people with secondary school education level accounted for the largest proportion. This result is also broadly consistent with Changchang Li ‘s study (Li et al., 2021), despite the different criteria for categorizing educational levels.

Results from the case-control study indicated that serum 25-(OH)D deficiency had OR values greater than 1, signifying a significant risk factor in both infected and healthy individuals, as well as cured and non-cured patients. Decreased serum vitamin D levels may increase the risk of C. t infection and treatment failure, aligning with previous laboratory findings. Although similar trends were observed in the 40–49 years age group, none of them were statistically significant, probably because of the small sample size. Analysis of the relationship between vitamin D levels and sex-based cure rates revealed that vitamin D levels were higher in males than in females in both healthy and infected populations, consistent with previous findings by Yan et al (Yan et al., 2017). This could be because Chinese women limit sun exposure and outdoor exercise to maintain fair skin, decreasing endogenous vitamin D synthesis. Cure rates were also higher in men than in women, similar to a prior study but not statistically significant (Kaushic et al., 2000). This phenomenon, attributed partly to the influence of estrogen and progesterone in women, may also be associated with lower vitamin D levels (Lee et al., 2009).

In the healthy population, the prevalence of vitamin D deficiency among participants of different ages and educational levels was around 50%, with no significant difference. This result is significantly lower than the 83% prevalence in China mentioned in the study by Jiang et al (Jiang et al., 2020). The reason for this may be related to the small sample size and the strict inclusion and exclusion criteria of this study.

In summary, this study reinforces the close association of vitamin D levels with C. t infection and treatment outcomes, suggesting that vitamin D deficiency may increase the risk of infection and treatment failure. Although our animal experiments demonstrated improved treatment success rates with vitamin D combined with antibiotic therapy than with antibiotic therapy alone, further validation through larger, prospective, randomized controlled trials is warranted to ascertain its potential as an adjunctive therapy alongside antibiotic therapy for C. t infections in clinical settings. However, given the global prevalence of vitamin D deficiency and the affordability and safety of vitamin D supplements, both healthy individuals and patients should prioritize vitamin D intake and supplementation.

## Data availability statement

The original contributions presented in the study are included in the article/**Supplementary Material**. Further inquiries can be directed to the corresponding author.

## Ethics statement

The studies involving humans were approved by Ethics Committee of Tianjin Medical University General Hospital. The studies were conducted in accordance with the local legislation and institutional requirements. The participants provided their written informed consent to participate in this study. The animal study was approved by Ethics Committee of Tianjin Medical University General Hospital. The study was conducted in accordance with the local legislation and institutional requirements.

## Author contributions

SL: Software, Validation, Visualization, Writing – original draft, Writing – review & editing. TZ: Methodology, Resources, Formal analysis, Investigation, Writing – original draft. QL: Resources, Writing – review & editing, Methodology, Project administration, Validation, Writing – original draft.
