# Supplementary material for: Vitamin D effects on Chlamydia trachomatis infection: a case-control and experimental study
Source: Front Cell Infect Microbiol. 2024 Apr 18;14:1366136. doi: 10.3389/fcimb.2024.1366136 (PMC11063265; doi:10.3389/fcimb.2024.1366136)
Supplement: Supplementary file 1 [file Image_1.pdf]

Supplementary Material

Supplementary Figure

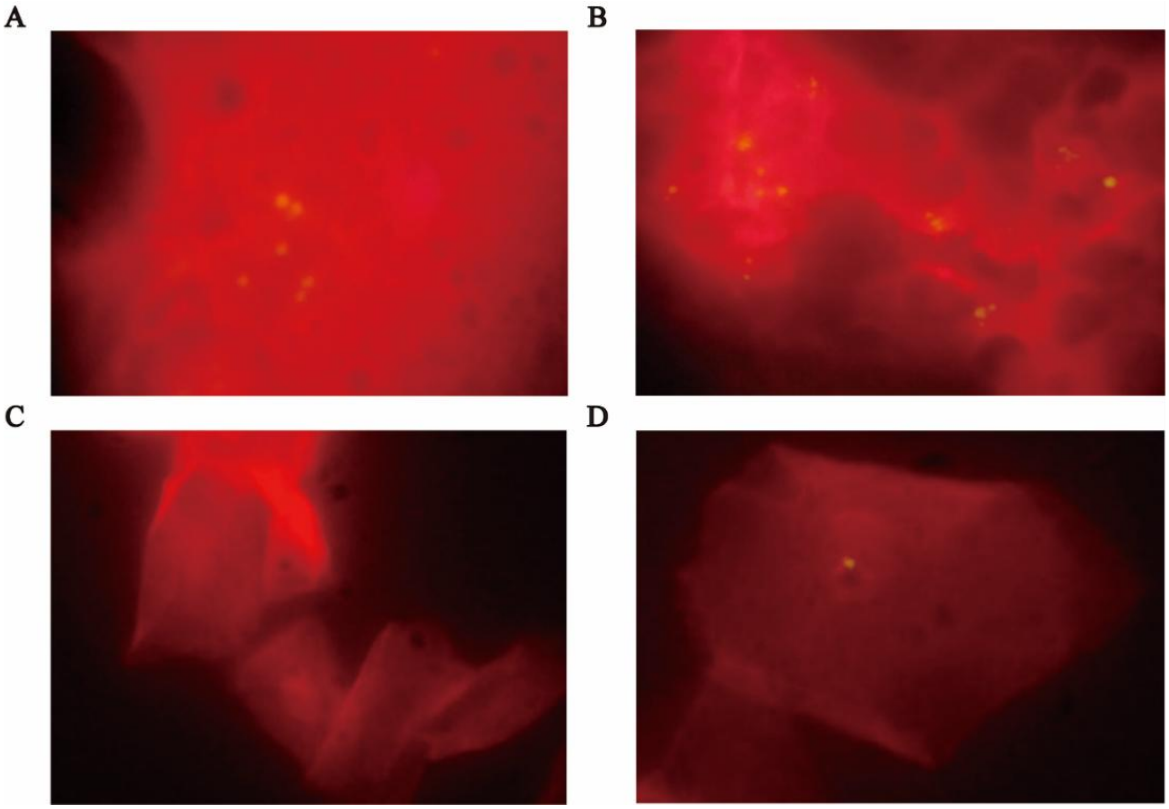

Supplementary Figure S1. Effect of 25-(OH)D level on C. t infection in the urogenital tract of mice. One week after infection, immunofluorescence staining of secretion smears from mice in the VD+ (A) and VD- (B) groups mice showed circular EBs emitting fluorescence. (C) The negative results of the secretion smears from mice in the VD+ group after 4 weeks of infection. (D) Fluorescent EBs remained visible in the secretions of mice in the VD- group 10 weeks after infection.
