# Supplementary material for: Vitamin D effects on Chlamydia trachomatis infection: a case-control and experimental study
Source: Front Cell Infect Microbiol. 2024 Apr 18;14:1366136. doi: 10.3389/fcimb.2024.1366136 (PMC11063265; doi:10.3389/fcimb.2024.1366136)
Supplement: Supplementary file 2 [file Table_1.docx]

Supplementary Material

## Supplementary Table

Supplementary Table S1. The serum 25-(OH)D levels of mice

| Mice | The serum 25-(OH)D levels (nmol/L) |
| --- | --- |
| VD+group_1 | 768.98 |
| VD+group_2 | 664.30 |
| VD+group_3 | 570.93 |
| VD+group_4 | 695.05 |
| VD+group_5 | 804.89 |
| VD+group_6 | 681.95 |
| VD+group_7 | 599.87 |
| VD+group_8 | 549.66 |
| VD+group_9 | 514.67 |
| VD+group_10 | 542.80 |
| VD+group_11 | 676.40 |
| VD+group_12 | 645.92 |
| VD+group_13 | 632.05 |
| VD+group_14 | 687.36 |
| VD+group_15 | 722.21 |
| VD-group_1 | 32.462 |
| VD-group_2 | 41.772 |
| VD-group_3 | 33.605 |
| VD-group_4 | 33.267 |
| VD-group_5 | 40.458 |
| VD-group_6 | 33.855 |
| VD-group_7 | 35.475 |
| VD-group_8 | 37.454 |
| VD-group_9 | 42.328 |
| VD-group_10 | 38.764 |
| VD-group_11 | 28.393 |
| VD-group_12 | 32.311 |
| VD-group_13 | 30.146 |
| VD-group_14 | 36.277 |
| VD-group_15 | 34.262 |
| VD-group_16 | 36.337 |
| VD-group_17 | 23.252 |
| VD-group_18 | 30.864 |
| VD-group_19 | 40.042 |
| VD-group_20 | 35.987 |
| VD-group_21 | 26.464 |
| VD-group_22 | 34.980 |
| VD-group_23 | 20.886 |
| VD-group_24 | 29.230 |
| VD-group_25 | 32.697 |
